# Effect of Different Levels and Sources of Dietary Copper, Zinc and Manganese on the Performance and Immune and Redox Status of Turkeys

**DOI:** 10.3390/ani9110883

**Published:** 2019-10-30

**Authors:** Jan Jankowski, Katarzyna Ognik, Krzystof Kozłowski, Anna Stępniowska, Zenon Zduńczyk

**Affiliations:** 1 Department of Poultry Science, Faculty of Animal Bioengineering, University of Warmia and Mazury, Oczapowskiego 5, 10-719 Olsztyn, Poland; janj@uwm.edu.pl (J.J.); kristof@uwm.edu.pl (K.K.); 2 Department of Biochemistry and Toxicology, Faculty of Animal Sciences and Bioeconomy, University of Life Sciences in Lublin, Akademicka 13, 20-950 Lublin, Poland; anna.stepniowska@up.lublin.pl; 3 Institute of Animal Reproduction and Food Research, Polish Academy of Sciences, Tuwima 10, 10-748 Olsztyn, Poland; z.zdunczyk@pan.olsztyn.pl

**Keywords:** copper, zinc, manganese, nanoparticles, turkeys

## Abstract

**Simple Summary:**

Companies producing turkey genetic (breeding) material recommend a much higher addition of Cu, Zn, and Mn to compound feed than recommended by the NRC. In practice, the poultry breeding companies in their feeding programs use an addition of these elements similar to the recommendations of breeding companies, without taking into account their content in the raw materials used for the production of feed. The aim of the study was to determine the effectiveness of the combined use of Cu, Zn, and Mn nanoparticles in the diet of slaughter turkeys with regard to potential reduction of the levels of these elements added to feed. Turkeys received the addition of Cu, Zn, and Mn in inorganic form or nanoparticles in amounts recommended by breeding companies, reduced to 10% of these recommendations or without the addition of these elements. The results of our research, showed that reducing the addition of these elements does not cause deterioration of performance and immunity of turkeys. It was also found that the addition of standard forms of these elements (inorganic forms) can be reduced without having to be replaced by nanoparticles.

**Abstract:**

The aim of the study was to determine the effectiveness of the combined use of Cu, Zn, and Mn nanoparticles in the diet of turkeys with regard to potential reduction of the levels of these elements added to feed. The experiment was carried out on turkeys’ hens assigned to four groups. Turkeys from the PC group received feed with the addition of inorganic forms of Cu, Zn, and Mn in the B.U.T. (British United Turkeys) recommended levels, from group IR received the addition in amounts reduced to 10% of the recommended levels, and from group NR received the addition of elements in the form of nanoparticles in the same amounts as in group IR. The turkeys from group NC received feed without the addition of these elements. The research showed that the addition of Cu, Zn, and Mn to turkey diets, in both inorganic forms and as nanoparticles, in quantities covering only 10% of B.U.T. recommendations had no adverse effect on growth performance or on the antioxidant and immune defense of turkeys. The changes in the redox status of the turkeys whose diet was not supplemented with Cu, Zn, and Mn indicate reduced oxidation processes in the tissues.

## 1. Introduction

Copper (Cu), zinc (Zn), and manganese (Mn) are micronutrients involved in numerous physiological processes, and they are essential for optimal health and growth of birds [1,2,3,4,5,6,7]. They play an important role stimulating the immune system [8,9], and additionally support the neutralization of free radicals, which cause serious cell damage [10]. Due to these positive functions, poultry diets are enriched with these elements, usually in their inorganic form [11,12]. 

According to the NRC (National Research Council) [13], Mn should be added to the diet of growing turkeys in the amount of 60 mg/kg of feed throughout the rearing period. Recommendations by B.U.T. (British United Turkeys) [14] are much higher—120 mg/kg of feed in the first four weeks of rearing and 100 mg/kg after the fourth week. According to the NRC [13], the Zn requirement for growing turkeys is 40–70 mg/kg diet, while the B.U.T. recommendation is 100 mg/kg. According to Hybrid commercial nutrient guidelines recommendations [15], the diets of young turkeys should be supplemented with up to 30 mg Cu/kg, which is significantly more than the previously recommended Cu dose of 8 mg/kg by NRC [13] as well as the rates recommended by the EU Commission’s Directorate for Health and Food Safety [16]. According to SCAN (Scientific Committee for Animal Nutrition) [16], the dietary inclusion levels of available Cu from organic sources should be up to 20 mg Cu/kg of feed, and the total Cu content of animal diets should not exceed 35 mg/kg. According to EU regulation 2018/1039, the maximum dose of Cu for all birds is 25 mg/kg [17].

The practice of adding inorganic forms of elements in amounts exceeding NRC [13] recommendations may interfere with the availability of other minerals [18,19,20], negatively affecting health [21,22], and resulting in excretion of large amounts of these elements into the environment, thereby contaminating it [8,10,23]. Increased awareness among producers and researchers of the effects of increased supplementation of poultry feed with trace elements has prompted the search for alternative solutions aimed at reducing the level of mineral supplementation without adverse consequences for the health and growth of birds [23]. Research carried out in the direction of looking for opportunities to reduce the addition of minerals to the poultry diet is of great practical and economic importance. It has been demonstrated that organic sources of trace elements are excreted into the environment to a lesser extent than inorganic forms, due to their greater bioavailability and stability in the upper digestive tract of poultry [24]. According to some researchers, however, despite the fact that organic forms can be used in smaller amounts than inorganic forms, the higher cost of using organic forms still discourages producers from using them [8,10]. Admittedly, Cu, Zn, or Mn nanoparticles are more expensive than their inorganic or even organic forms, but, with the assumption that due to their high biological activity, it will be possible to use much smaller amounts of these microelements in the diet (while maintaining good performance), the cost will be comparable. Many studies, summarized in a review by Scott et al. [10], indicate that the addition of Cu, Zn, and Se nanoparticles to the diet favorably stimulates the immune system, antioxidant system, and growth of birds. According to Sri Sindhura et al. [25], the small size of nanoparticles increases their biological activity. Therefore, the amount of nanoparticles added to the diet may be reduced more than commonly used elements with the size of macromolecules. However, little research has been done to determine the effectiveness of Cu, Zn, and Mn nanoparticles added together to feed for poultry, particularly turkeys. We hypothesized that nanoparticles, due to their high physical reactivity, can be used as an alternative, effective promoter of health and growth of turkeys in much smaller amounts than inorganic forms of these elements, thereby significantly reducing the excretion of these minerals into the environment.

The aim of the study was to determine the effectiveness of the combined use of Cu, Zn, and Mn nanoparticles in the diet of turkeys with regard to potential reduction of the levels of these elements added to feed.

## 2. Material and Methods

### 2.1. Animals

The experimental procedure was approved by the Local Ethics Committee for Experiments with Animals in Olsztyn, Poland (approval no. 30/2015). The experiment was carried out on one-day old Hybrid Converter turkey hens, purchased at the Grelavi turkey hatchery in Kętrzyn. Turkeys were kept until 112 days of age. A total of 1400 turkeys were allocated to 28 pens (50 turkeys/10 m^2^) at the Teaching and Research Laboratory in Bałdy near Olsztyn, Poland. The experiment was set up in a completely randomized design with four groups. Feeding groups within the experiment comprised seven pens with 50 birds, each of which constituted a replication within the group. Replications (pens) were assigned to groups in a way that ensured that they were evenly distributed in the building. The stocking density at the initial stage of rearing was 5 birds/m^2^. The ambient conditions, regulated automatically, were adjusted to the age of the birds and to Hybrid recommendations and were the same for all birds in the experimental building. Throughout the experiment, all birds had unlimited access to the diet and water. The height of the drinkers and feeders was adjusted to the stage of growth. Turkeys from the PC group received feed with the addition of inorganic forms of elements in the following amounts: 20 mg Cu (CuSO_4_), 100 mg Zn (ZnO), and 100 mg Mn (MnO)/kg, i.e., 100% coverage of the B.U.T. nutritional recommendations (Hybrid Turkeys, 2013), most often applied in practical feeding of turkeys. The turkeys from group IR received feed with the addition of inorganic forms of elements in amounts reduced to 10% of the recommended levels: 2 mg Cu, 10 mg Zn, and 10 mg Mn/kg. The turkeys in group NR received feed with the addition of elements in the form of nanoparticles in the same amounts as in group IR. The turkeys from group NC received feed without the addition of these elements. The composition of the basal diets, their calculated nutritional value, and the periods they were used are presented in Table 1. The diets were given in the form of crumbles (1–28 days) or pellets (from day 29). Manganese nanopowder (NP-Mn_2_O_3_, purity 98%, 40–60 nm, spherical specific surface area 13.5 m^2^/g, bulk density 1.2 g/cm^3^), copper nanoparticles (NP-Cu, 25 nm, in the form of 99.8% purity powder), and zinc nanoparticles (NP-ZnO, 25 nm, in the form of 99.8% purity powder), were purchased from SkySpring Nanomaterials (City, US State abbrev., USA). The diets were produced by Agrocentrum sp. z o.o. in two stages: (1) as basal feeds without the addition of a vitamin-mineral premix, and then (2) with vitamin mineral premixes containing the appropriate amount and form of Mn, Cu and Zn added to the feed for each experimental group (Table 1).

### 2.2. Growth Trial and Sample Collection

During the experiment, body weight (BW) and feed intake (FI) were recorded and calculated on a pen basis. Daily feed intake (DFI) per bird was calculated based on total feed intake per pen during the entire experimental period divided by the number of days in the period. Feed conversion ratio (FCR) was calculated on a pen basis from body weight gain and feed intake. 

At the age of 112 days, 14 birds representing the average body weight of each group were selected and tagged. Blood samples were taken from the wing vein of 14 tagged birds from each group (two birds for each replication) with BW similar to the treatment average. The birds were slaughtered in a processing plant 8 h after feed withdrawal (slaughterhouse of the Faculty of Animal Bioengineering, University of Warmia and Mazury, Olsztyn, Poland). The slaughterhouse equipment and all procedures were approved by the Local Animal Care and Use Committee (Olsztyn, Poland; permit number 30/2015). The birds were electrically stunned (400 mA, 350 Hz), hung on a shackle line, and exsanguinated by a unilateral neck cut severing the right carotid artery and jugular vein. After slaughter, the carcasses were scalded, plucked, and eviscerated; subsequently, they were stored at 4 °C and hand-deboned on a cone 24 h post mortem. The yields of whole carcass, breast muscles, leg muscles (thigh and drumstick without skin), heart, liver, gizzard, abdominal fat, spleen, bursa of Fabricius, femur, and tibia were determined relative to live BW. The redox status of the jejunum, liver, and bursa of Fabricius was determined in all samples collected from each group.

### 2.3. Laboratory Analysis

Diet, blood plasma, liver, breast muscle, and skin samples were analyzed for Cu, Zn, and Mn by inductively coupled plasma optical emission spectrometry. The concentration of lipid hydroperoxides (LOOH) and malondialdehyde (MDA) were determined in the blood as markers of redox and immune status, using kits produced by Cell Biolabs, Inc. (San Diego, CA, USA). Activity of the enzymes superoxide dismutase (Mn-SOD) and glutathione peroxidase (GPx) in the blood of the turkeys was determined by spectrometry using Ransel and Ransod diagnostic kits manufactured by Randox (Warszawa, Poland). A diagnostic kit manufactured by Oxis International, Inc. (Portland, ME, USA) was used to determine catalase activity (CAT). Activity of ceruloplasmin (Cp) in the blood plasma was determined using a Ceruloplasmin ELISA kit (Biomatik, Wilmington, DE, USA). Also determined in the blood were total glutathione (GSH+GSSG) using a Total Glutathione Assay (Cell Biolabs, Inc., San Diego, CA, USA), Caspase 3 and Caspase 8, and vitamin C content using an ELISA kit (Cell Biolabs, Inc., San Diego, CA, USA). Immunoglobulin IgM and interleukin (IL-6) in the blood were determined in an ELISA reader using assays from Elabscience Biotechnology Co., Ltd. (Houston, TX, USA). As described previously [26], the following indicators of redox status were determined in the jejunum, liver, breast muscle, and bursa of Fabricius: the concentrations of lipid hydroperoxides (LOOH) and malondialdehyde (MDA), total glutathione (GSH+GSSG), activity of superoxide dismutase (SOD) and catalase (CAT), content of vitamin C, and total antioxidant status (FRAP). 

### 2.4. Statistical Analysis

The data were subjected to statistical analysis using one-way analysis of variance. The significance of differences was verified by the Tukey test. All calculations were performed using the GLM (General Linear Model) procedures of the STATISTICA software system ver. 13.1 (StatSoft Inc., Kraków, Poland, 2016). Data variability was expressed as mean values with a pooled standard error of the mean (SEM), and *p* < 0.05 was considered statistically significant.

## 3. Results

The chemical analysis showed that 1 kg of the basal diets, without the addition of the test elements, contained 6–13 mg Cu, 39–78 mg Zn and 48–73 mg Mn, depending on the feeding period and thus on the feed composition (Table 1). In groups PC–NR, the content of these elements was in line with expectations, and the differences may have been due to the accuracy of the chemical analyses. Table 2 presents the growth performance of the turkeys in periods in which different experimental feeds were used during the entire experiment. Reducing or eliminating the addition of Cu, Zn, and Mn to the diet did not affect the turkey performance results. The final BW of turkeys and the FCR for the entire period were close to hybrid standards [15]. The survival rate of turkeys in all groups was very high, exceeding 97%. 

The dressing percentage, percentage of breast and leg muscles were similar in all groups. Reducing or eliminating the addition of Cu, Zn, and Mn to the diet resulted in a tendency to increase abdominal fat (*p* = 0.079). Supplementation of the basal diet with elements in the form of nanoparticles at a level reduced to 10% of recommendations (group NR) resulted in a decrease in relative liver weight (*p* = 0.031) as compared to turkeys from group PC and IR. Turkeys from group NC, which received feed without the addition of Cu, Zn, and Mn, had a lower relative gizzard weight (*p* = 0.033) compared to PC, IR, and NR, and greater spleen weight (*p* = 0.028) compared to PC and NR. In the NR group, there was lower relative femur weight compared to the IR and NC groups (*p* = 0.039) (Table 3).

Plasma Cu, Zn, and Mn levels were similar in all experimental groups. Compared to group PC, which received the recommended amount of Cu, Zn, and Mn, Mn content was higher in the liver from groups IR, NR, and NC (*p* < 0.001) and in the skin of turkeys from groups NR and NC (*p* < 0.001). In the skin of turkeys from groups IR and NR, the Cu content was lower (*p* < 0.001) than in group PC and NC, and in the skin of turkeys from groups IR, NR and NC, the Zn content was lower (*p* = 0.006) than in group PC. The use of Cu, Zn, and Mn in an amount reduced to 10% of the recommended level (group IR) caused an increase in the content of Zn (*p* = 0.022) in the liver relative to group PC and NR (Table 4). 

The use of Cu, Zn, and Mn in the turkey diets in amounts covering only 10% of the BUT recommendations for these nutrients (group IR) caused an increase in the plasma IL-6 level (*p* < 0.001) compared to group PC, NR, and NC (Table 5). 

In the plasma of turkeys from group NR, SOD activity was lower (*p* = 0.005) than in group PC, IR, and NC. GPx activity in turkey blood was higher in the IR group compared to the NC group (*p* = 0.016). The plasma content of GSH+GSSG in the turkeys from groups IR, NR, and NC was higher (*p* = 0.001) than in group PC. Turkeys fed IR had higher plasma vitamin C (*p* = 0.016) compared to turkeys fed PC and NR. In the blood plasma of turkeys from the IR group, lower LOOH levels were noted than in the NR and NC groups (*p* = 0.011) (Table 6). 

Higher SOD activity (*p* = 0.013) and lower MDA content (*p* < 0.001) in the small intestinal wall and higher LOOH content (*p* = 0.024) in the breast muscle were found in turkeys whose diet was not supplemented with Cu, Zn, and Mn (group NC) compared to group (PC) receiving the recommended levels of these minerals (Table 7 and Table 8). 

The use of Cu, Zn, and Mn in amounts reduced to 10% of the recommended levels in the turkey diet (group IR) led to a reduction in the content of MDA (*p* = 0.001) in the liver relative to the others groups (Table 9). 

Compared to group PC, receiving the recommended amount of Cu, Zn, and Mn, SOD activity in the bursa of Fabricius was lower (*p* < 0.001) in the turkeys for which the addition of these elements was reduced to 10%, both in the same forms (group IR) and in the form of the nanoparticles (group NR), as well as in the turkeys whose diet was not supplemented with these elements (group NC) (Table 10).

## 4. Discussion

In the present study, growth performance of turkeys was not shown to be negatively affected by the use of a diet with no addition of Cu, Zn, and Mn or by the addition of these elements (irrespective of form) in amounts covering only 10% of B.U.T. nutritional recommendations. These results are consistent with those of our previous studies, which found that reducing addition of Cu and Mn to the turkey diet even to 10% of B.U.T. recommendations had no negative effect on growth performance [3,6,7]. Similarly, Yang et al. [27] found no deterioration in the growth performance of chickens whose diet was not supplemented with inorganic forms of Cu, Zn, and Mn. Research by El-Husseiny et al. [28] and Gheisari et al. [29] have demonstrated that a 50% reduction of the Cu, Zn, and Mn supplementation recommended by NRC [13] for chicken diets improves growth performance. There are reports indicating that the addition of Zn in the form of nanoparticles covering only 10% or 20% of recommendations for this element has improved growth performance in chickens, whereas increasing Zn to a level covering 40% of nutritional recommendations did not produce this effect [30]. Reduction of Zn addition to turkey diet from 100 to 50 and 10 mg/kg [31], as well as the lack of Zn addition to chickens diet [32] was not found to affect growth performance. Azad et al. [33] reported that a lack of Zn supplementation had an adverse effect on rearing results.

In our study, lack of supplementation of turkey diets with Cu, Zn, and Mn caused a decrease in the relative weight of the gizzard. The addition of Cu, Zn, and Mn nanoparticles in amounts covering 10% of B.U.T. recommendations reduced the relative weight of the liver. Our previous research on turkeys showed that the addition of Mn to the turkey diet at 10% of B.U.T. recommendations, with Cu and Zn added at 100% of the recommended level, increased the relative weight of the liver [7]. An increase in the relative liver weight and improvement in the dressing percentage of chickens have been reported by El-Husseiny et al. [28], who gave chickens a diet supplemented with organic forms of Cu, Zn, and Mn in amounts 50% lower than the levels recommended by NRC [13].

In our research, reducing the addition of Cu, Zn, and Mn (irrespective of form) to the turkey diet resulted in a decrease in the content of Cu and Zn but an increase in the content of Mn in the skin. In the liver, the reduction in the addition of Cu, Zn, and Mn to the diet resulted in increased content of Zn and Mn. Changes in trace mineral absorption in the gut and excretion are the primary mechanisms for maintaining mineral homeostasis in the biological system. However, a higher level of one element may cause antagonism with another, reducing its absorption and increasing its excretion [34]. Our previous research found that reducing the addition of Mn to the diet of chickens 10-fold with respect to B.U.T. recommendations resulted in a decrease in intestinal absorption of this element, but only by 2%, whereas intestinal absorption of Zn was improved [7]. At the same time, our previous research showed that, irrespective of the form of Mn, reducing supplementation with this element resulted in greater accumulation in the liver, breast muscle, and skin [7]. According to Berta et al. [35] and Cholewińska et al. [36], increasing dietary supplementation with an element does not always increase its accumulation in all tissues proportionately because the excess can be absorbed and excreted. In addition, in response to reduced supplementation of minerals relative to the demand, the body may develop an adaptive mechanism (e.g., increased mobilization of transport proteins) to increase the deposition of selected minerals in the tissues [37,38]. Therefore, our results do not confirm those of El-Husseiny et al. [28], Aksu et al. [23], and Gajula et al. [39], who found that reducing the addition of Cu, Zn, and Mn to the chicken diet reduces the content of these minerals (especially Zn and Mn) in the organ tissues and bones.

Some experiments on chickens have shown that diet supplementation with higher levels of Cu, Zn, and Mn than recommended by NRC [13] increases the birds’ immunity [39,40]. In our research, addition of Cu, Zn, and Mn to the turkey diets in quantities covering only 10% of B.U.T. recommendations [15] had no effect on the level of the antibodies tested, resulting only in an increase in the plasma level of IL-6. However, it is difficult to interpret this result as an increased inflammatory reaction caused by a deficiency of these elements, as the complete lack of any supplementation with Zn, Mn, and Cu did not cause an increase in this cytokine. Cell-mediated immunity is closely associated with the production of cytokines. However, an increase in the level of cytokines may also occur due to increased Zn or Mn supplementation [39]. In our other research on turkeys, reduction of the level of Mn added to the diet of young turkeys caused an increase in plasma IL-6 [7]. On the other hand, in research on chickens, a diet containing 11% less Cu than recommended by NRC [13] did not cause an increase in plasma IL-6, whereas increasing the level of Cu in the diet to exceed NRC recommendations resulted in an increase in plasma IL-6 levels [4].

In the present study, the lack of added Cu, Zn, and Mn, as well as the addition of these elements (irrespective of form) in amounts covering only 10% of B.U.T. recommendations, caused an increase in the plasma content of GSH+GSSG in the turkeys. The lack of Cu, Zn, and Mn supplementation in the turkey diet was also found to reduce the severity of oxidative processes, as indicated by the reduced MDA content and by the increased SOD activity in the intestinal wall. Similar results were obtained in our earlier study on turkeys, in which Cu at 20 mg/kg induced oxidation reactions and had a much stronger inhibitory effect on the antioxidant defense system than dietary Cu at 2 mg/kg [3]. Moreover, research on chickens indicates that reducing the addition of Cu to the diet limits oxidative processes in the body [41,42,43]. In our previous research, reducing the addition of Mn nanoparticles to the diet of young turkeys (reared up to six weeks of age) to 50% or even 10% of the level recommended by B.U.T. had no adverse effect on antioxidant defense, whereas reducing the addition of Mn in the form of MnO intensified lipid oxidation processes [7]. In our research on turkeys reared for a longer period (up to 15 weeks of age) and receiving diets supplemented with Mn in the amount of 50% and 10% of the level recommended by B.U.T., oxidation of lipids, proteins, and DNA was found to increase in proportion to the decrease in Mn [5]. The results of the present study regarding the redox status of turkeys are not in agreement with those obtained in our other research [5,6,7], in which an attempt was made to reduce the addition of Mn to the turkey diet. In the present study, the addition of three elements, Cu, Zn, and Mn, was simultaneously reduced or eliminated, while, in our previous study [5], only the addition of Mn to the turkey diet was reduced, while Zn and Cu were provided according to B.U.T. guidelines. These differences in the treatments could unquestionably have led to different results. El-Husseiny et al. [28], in a study on chickens receiving Cu, Zn, and Mn, found that the proportions of these elements in the diet significantly influenced their accumulation in tissues, growth performance, carcass analysis, and even immunity.

## 5. Conclusions

The research showed that the addition of Cu, Zn, and Mn to turkey diets, in both conventional forms and as nanoparticles, in quantities covering only 10% of B.U.T. recommendations had no adverse effect on growth performance or on the antioxidant and immune defense of turkeys. A lack of added Cu, Zn, and Mn in the turkey diet also did not negatively affect growth performance or immunity, and even reduced the content of MDA in the wall of the small intestine and in the liver. The changes in the redox status of the turkeys whose diet was not supplemented with Cu, Zn, and Mn may indicate reduced oxidation processes in the tissues.

## Figures and Tables

**Table 1:** Composition and nutrient density of diets.

	Feeding Period, Days			
Components, g/kg	1–28	29–56	57–84	85–112
Wheat	521.6	547.6	650.6	744.3
Soybean meal	409.5	354.1	236.5	143.5
Rapeseeds	-	20.0	40.0	60.0
Soybean oil	18.9	31.6	33.7	20.9
MCP	16.4	15.5	9.8	5.1
Limestone	16.6	14.7	13.7	12.1
Salt	2.0	1.6	1.6	1.2
L-Lysine HCl (780 g/kg)	4.6	4.5	4.6	4.3
DL-Methionine (990 g/kg)	2.9	2.7	1.8	1.4
L-Threonine (985 g/kg)	1.0	1.2	1.2	0.7
NaHCO_3_	1.5	1.5	1.5	1.5
Min-vit. Premix ^1^	5.0	5.0	5.0	5.0
Calculated nutrient density, g/kg				
Crude protein	265.0	240.0	205.0	175.0
Calcium	11.5	11.0	8.5	6.5
Available phosphorus	5.5	5.0	4.0	3.0
Sodium	1.5	1.3	1.3	1.1
Lysine	17.0	15.5	13.0	10.7
Met+cys	11.0	10.0	8.5	7.5
Threonine	10.3	9.6	8.2	6.5
AME (kcal/kg)	2750	2900	3050	3100
Amount of Cu added to feed	Analysed content of Cu, mg/kg			
20 mg/kg CuSO_4_ (PC)	24	31	28	27
2 mg/kg CuSO_4_ (IR)	12	15	12	8
2 mg/kg NP-Cu (NR)	10	16	14	7
0 mg/kg (NC)	10	13	10	6
Amount of Zn added to feed	Analysed content of Zn, mg/kg			
100 mg/kg ZnO (PC)	125	140	139	138
10 mg/kg ZnO (IR)	49	79	64	58
10 mg/kg NP-ZnO (NR)	61	84	82	49
0 mg/kg (NC)	50	78	55	39
Amount of Mn added to feed	Analysed content of Mn, mg/kg			
100 mg/kg MnO (PC)	124	113	146	141
10 mg/kg MnO (IR)	57	86	78	62
10 mg/kg NP-Mn_2_O_3_ (NR)	68	81	95	53
0 mg/kg (NC)	57	73	70	48

^1^ Mineral-vitamin premix per kilogram of diet (without Cu, Zn, and Mn supplement): vitamin A—15,000 IU; vitamin D_3_—5000 IU; vitamin E—100 IU; vitamin K_3_–4 mg; vitamin B_1_—5 mg; vitamin B_2_—15 mg; vitamin B_6_—6 mg; vitamin B_12_—0.04 mg; nicotinic acid—100 mg; calcium pantothenicum—32.7 mg; folic acid—4 mg; choline—700 mg; biotin—0.35 mg; Se—0.3 mg; Fe—60 mg; I—1.5 mg; Ca—1.04 g. PC—control group receiving a mineral and vitamin premix containing (per kg of feed): 20 mg CuSO_4_, 100 mg ZnO and 100 mg MnO; IR—group receiving a mineral and vitamin premix containing (per kg of feed): 2 mg CuSO_4_, 10 mg ZnO and 10 mg MnO; NR—group receiving a mineral and vitamin premix containing (per kg of feed): 2 mg NP-Cu, 10 mg NP-ZnO and 10 mg NP-Mn_2_O_3_; NC—group receiving a mineral and vitamin premix without Cu, Zn, or Mn.

**Table 2:** Growth performance of turkeys.

Item	PC	IR	NR	NC	SEM	*p*-Value
BW, 1 day kg	0.065	0.065	0.065	0.065	<0.001	0.570
BW, 28 days kg	1.254	1.257	1.261	1.249	0.004	0.818
BW, 56 days kg	4.094	4.086	4.096	4.044	0.015	0.614
BW, 84 days kg	7.862	7.872	7.825	7.739	0.024	0.181
BW, 112 days kg	10.43	10.41	10.43	10.40	0.031	0.992
BWG, 1–28 days g/day	42.5	42.6	42.7	42.3	0.155	0.822
BWG, 29–56 days g/day	101.3	101.0	101.3	99.8	0.489	0.669
BWG, 57–84 days g/day	134.5	135.2	133.2	132.0	0.655	0.314
BWG, 85–112 days g/day	91.8	90.9	93.1	95.3	0.832	0.277
BWG, 1–112 days g/day	92.6	92.4	92.6	92.3	0.279	0.992
DFI, 1–28 days g/bird/day	61.5	61.7	61.7	61.4	0.170	0.952
DFI, 29-56 days g/bird/day	171.6	173.4	174.9	170.7	0.980	0.452
DFI, 57–84 days g/bird/day	339.7	339.6	342.7	338.7	1.780	0.874
DFI, 85–112 days g/bird/day						
DFI, 1–112 days g/bird/day	238.2	243.4	241.1	235.6	1.425	0.240
FCR, 1–28 days kg/kg	1.448	1.450	1.444	1.453	0.005	0.936
FCR, 29–56 days kg/kg	1.685	1.713	1.722	1.703	0.009	0.535
FCR, 57–84 days kg/kg	2.538	2.524	2.574	2.571	0.010	0.261
FCR, 85–112 days kg/kg	3.931	3.862	3.883	3.774	0.027	0.236
FCR, 1–112 days kg/kg	2.500	2.501	2.532	2.503	0.006	0.195
Liveability %	97.1	97.1	99.4	98.6	0.488	0.268

BWG—body weight gain; BW—body weight; DFI—daily feed intake, FCR—feed conversion ratio; PC—control group receiving a mineral and vitamin premix containing (per kg of feed): 20 mg CuSO_4_, 100 mg ZnO and 100 mg MnO; IR—group receiving a mineral and vitamin premix containing (per kg of feed): 2 mg CuSO_4_, 10 mg ZnO and 10 mg MnO; NR—group receiving a mineral and vitamin premix containing (per kg of feed): 2 mg NP-Cu, 10 mg NP-ZnO and 10 mg NP-Mn_2_O_3_; NC—group receiving a mineral and vitamin premix without Cu, Zn, or Mn.

**Table 3:** Results of turkey carcass analysis and relative weights of selected organs and bones (body weight = 100%).

Item	PC	IR	NR	NC	SEM	*p*-Value
Carcass yield, %	80.50	80.58	81.26	81.26	0.219	0.459
Breast muscles, %	23.59	22.80	22.26	23.30	0.249	0.252
Thigh muscles, %	10.05	10.42	9.92	10.34	0.113	0.371
Drumstick muscles, %	7.65	8.00	7.80	8.28	0.105	0.161
Abdominal fat, %	1.46 ^C^	1.61 ^B^	1.86 ^A^	1.89 ^A^	0.069	0.079
Gizzard, %	0.75 ^a^	0.75 ^a^	0.72 ^a^	0.62 ^b^	0.018	0.033
Liver, %	1.36 ^a^	1.40 ^a^	1.18 ^b^	1.29 ^ab^	0.030	0.031
Heart, %	0.309	0.281	0.268	0.277	0.006	0.062
Spleen, %	0.091 ^b^	0.123 ^ab^	0.069 ^b^	0.150 ^a^	0.011	0.028
Bursa of Fabricius, %	0.070	0.059	0.069	0.067	0.195	0.199
Femur, %	0.454 ^ab^	0.480 ^a^	0.446 ^b^	0.487 ^a^	0.006	0.039
Tibia, %	0.583	0.613	0.564	0.595	0.007	0.103

a, b—means within the same row differ significantly (*p* ≤ 0.05); A–C values in the same row with no common superscripts show a near significant trend (0.05 < *p* < 0.10); PC—control group receiving a mineral and vitamin premix containing (per kg of feed): 20 mg CuSO_4_, 100 mg ZnO and 100 mg MnO; IR—group receiving a mineral and vitamin premix containing (per kg of feed): 2 mg CuSO_4_, 10 mg ZnO and 10 mg MnO; NR—group receiving a mineral and vitamin premix containing (per kg of feed): 2 mg NP-Cu, 10 mg NP-ZnO and 10 mg NP-Mn_2_O_3_; NC—group receiving a mineral and vitamin premix without Cu, Zn, and Mn.

**Table 4:** Content of Mn, Cu, and Zn in turkey tissues.

Item		PC	IR	NR	NC	SEM	*p*-Value
Plasma	Mn mg/L	LOQ	LOQ	LOQ	LOQ	-	-
	Cu µmol/L	3.01	3.06	3.39	3.09	0.118	0.675
	Zn µmol/L	28.21	28.66	31.81	28.96	1.108	0.675
Liver	Mn mg/kg	2.477 ^d^	3.105 ^c^	3.660 ^b^	4.460 ^a^	0.170	<0.001
	Cu mg/kg	2.730	3.385	2.608	2.973	0.136	0.193
	Zn mg/kg	19.87 ^b^	23.95 ^a^	20.98 ^b^	22.37 ^ab^	0.521	0.022
Breast muscle	Mn mg/kg	0.545	0.464	0.362	0.611	0.041	0.162
	Cu mg/kg	2.513	2.817	2.722	3.167	0.124	0.319
	Zn mg/kg	9.607	9.245	9.897	10.008	0.316	0.851
Skin	Mn mg/kg	0.348 ^c^	0.513 ^c^	1.232 ^b^	2.147 ^a^	0.161	<0.001
	Cu mg/kg	0.669 ^a^	0.525 ^b^	0.533 ^b^	0.777 ^a^	0.028	<0.001
	Zn mg/kg	10.427 ^a^	6.230 ^b^	7.610 ^b^	7.728 ^b^	0.468	0.006

a, b, c—means within the same row differ significantly (*p* ≤ 0.05); LOQ ˂ 0.04 mg/L; PC—control group receiving a mineral and vitamin premix containing (per kg of feed): 20 mg CuSO_4_, 100 mg ZnO and 100 mg MnO; IR—group receiving a mineral and vitamin premix containing (per kg of feed): 2 mg CuSO_4_, 10 mg ZnO and 10 mg MnO; NR—group receiving a mineral and vitamin premix containing (per kg of feed): 2 mg NP-Cu, 10 mg NP-ZnO and 10 mg NP-Mn_2_O_3_; NC—group receiving a mineral and vitamin premix without Cu, Zn, and Mn.

**Table 5:** Indicators of immune status and apoptosis in turkeys.

Item	PC	IR	NR	NC	SEM	*p*-Value
IgM, ng/mL	453.56	471.67	512.24	466.51	13.451	0.465
IL-6, pg/mL	9.59 ^b^	12.04 ^a^	9.66 ^b^	8.75 ^b^	0.276	<0.001
Casp 3, pg/mL	54.42	61.52	58.77	60.40	1.185	0.156
Casp 8, ng/mL	7.34	7.76	7.79	8.56	0.212	0.232

a, b—means within the same row differ significantly (*p* ≤ 0.05); Casp 3—caspase 3; Casp 8—caspase 8; IgM—immunoglobulin M; IL-6—interleukin 6; PC—control group receiving a mineral and vitamin premix containing (per kg of feed): 20 mg CuSO_4_, 100 mg ZnO and 100 mg MnO; IR—group receiving a mineral and vitamin premix containing (per kg of feed): 2 mg CuSO_4_, 10 mg ZnO and 10 mg MnO; NR—group receiving a mineral and vitamin premix containing (per kg of feed): 2 mg NP-Cu, 10 mg NP-ZnO and 10 mg NP-Mn_2_O_3_; NC—group receiving a mineral and vitamin premix without Cu, Zn, and Mn.

**Table 6:** Indicators of redox status in turkey blood.

Item	PC	IR	NR	NC	SEM	*p*-Value
Cp, U/L	1.095	1.298	1.298	1.035	0.060	0.288
SOD, U/gHb	200.46 ^a^	205.24 ^a^	141.64 ^b^	216.21 ^a^	8.598	0.005
GPx, U/g Hb	37.04 ^ab^	43.39 ^a^	36.64 ^ab^	30.78 ^b^	1.455	0.016
CAT, U/gHb	1788.3	1849.5	1628.6	1792.0	54.811	0.543
FRAP, µmol/L	123.9	121.3	117.7	113.2	1.502	0.059
GSH+GSSG, µmol/L	0.136 ^b^	0.214 ^a^	0.211 ^a^	0.205 ^a^	0.009	0.001
VIT C, µmol/L	89.29 ^b^	100.19 ^a^	92.13 ^b^	96.18 ^ab^	1.340	0.016
LOOH, µmol/L	11.51 ^ab^	9.88 ^b^	12.46 ^a^	12.88 ^a^	0.364	0.011
MDA, µmol/L	1.600	1.834	1.644	1.834	0.089	0.715

a, b—means within the same row differ significantly (*p* ≤ 0.05); SOD—superoxide dismutase, GPx—glutathione peroxidase; CAT—catalase; Cp—ceruloplasmin; GSH+GSSG—total glutathione; VIT C—vitamin C; LOOH—lipid peroxides; MDA—malondialdehyde; FRAP—total antioxidant status; PC—control group receiving a mineral and vitamin premix containing (per kg of feed): 20 mg CuSO_4_, 100 mg ZnO and 100 mg MnO; IR—group receiving a mineral and vitamin premix containing (per kg of feed): 2 mg CuSO_4_, 10 mg ZnO and 10 mg MnO; NR—group receiving a mineral and vitamin premix containing (per kg of feed): 2 mg NP-Cu, 10 mg NP-ZnO and 10 mg NP-Mn_2_O_3_; NC—group receiving a mineral and vitamin premix without Cu, Zn, and Mn.

**Table 7:** Indicators of redox status in the small intestinal wall of turkeys.

Item	PC	IR	NR	NC	SEM	*p*-Value
SOD U/g protein	6.73 ^b^	5.93 ^b^	7.01 ^b^	8.63 ^a^	0.314	0.013
CAT U/g protein	44.08	40.79	40.57	41.47	0.875	0.483
GSH+GSSG µmol/kg	0.450	0.430	0.420	0.451	0.012	0.763
VIT C µmol/kg	72.85	80.03	76.42	80.63	1.418	0.184
LOOH µmol/ kg	15.85	15.11	14.15	15.00	0.406	0.554
MDA µmol/kg	8.30 ^ab^	7.09 ^b^	8.98 ^a^	5.09 ^c^	0.355	<0.001

a, b, c—means within the same row differ significantly (*p* ≤ 0.05); SOD—superoxide dismutase; CAT—catalase; GSH+GSSG—total glutathione; VIT C—vitamin C; LOOH—lipid peroxides; MDA—malondialdehyde; PC—control group receiving a mineral and vitamin premix containing (per kg of feed): 20 mg CuSO_4_, 100 mg ZnO and 100 mg MnO; IR—group receiving a mineral and vitamin premix containing (per kg of feed): 2 mg CuSO_4_, 10 mg ZnO and 10 mg MnO; NR—group receiving a mineral and vitamin premix containing (per kg of feed): 2 mg NP-Cu, 10 mg NP-ZnO and 10 mg NP-Mn_2_O_3_; NC—group receiving a mineral and vitamin premix without Cu, Zn, and Mn.

**Table 8:** Indicators of redox status in the breast muscle of turkeys.

Item	PC	IR	NR	NC	SEM	*p*-Value
SOD U/g protein	6.56	5.71	6.95	6.14	0.269	0.408
CAT U/g protein	11.57	11.14	10.00	11.35	0.479	0.684
GSH+GSSG µmol/kg	0.223	0.239	0.227	0.241	0.003	0.163
VIT C µmol/kg	82.30	83.69	83.45	81.05	1.037	0.811
LOOH µmol/ kg	2.508 ^b^	2.464 ^b^	2.713 ^ab^	2.930 ^a^	0.063	0.024
MDA µmol/kg	1.903	1.802	1.820	1.792	0.025	0.395

a, b—means within the same row differ significantly (*p* ≤ 0.05); SOD—superoxide dismutase; CAT—catalase; GSH+GSSG—total glutathione; VIT C—vitamin C; LOOH—lipid peroxides; MDA—malondialdehyde; PC—control group receiving a mineral and vitamin premix containing (per kg of feed): 20 mg CuSO_4_, 100 mg ZnO and 100 mg MnO; IR—group receiving a mineral and vitamin premix containing (per kg of feed): 2 mg CuSO_4_, 10 mg ZnO and 10 mg MnO; NR—group receiving a mineral and vitamin premix containing (per kg of feed): 2 mg NP-Cu, 10 mg NP-ZnO and 10 mg NP-Mn_2_O_3_; NC—group receiving a mineral and vitamin premix without Cu, Zn, and Mn.

**Table 9:** Indicators of redox status in the liver of turkeys.

Item	PC	IR	NR	NC	SEM	*p*-Value
SOD U/g protein	4.67	5.83	5.19	5.82	0.235	0.241
CAT U/g protein	58.07	51.40	57.01	58.21	1.322	0.218
GSH+GSSG µmol/kg	0.378	0.393	0.449	0.445	0.017	0.329
VIT C µmol/kg	170.58	173.45	167.85	168.04	4.178	0.965
LOOH µmol/ kg	6.83	6.83	6.82	6.84	0.005	0.630
MDA µmol/kg	5.71 ^a^	4.66 ^b^	6.36 ^a^	6.02 ^a^	0.174	0.001

a, b—means within the same row differ significantly (*p* ≤ 0.05); SOD—superoxide dismutase; CAT—catalase; GSH+GSSG—total glutathione; VIT C—vitamin C; LOOH—lipid peroxides; MDA—malondialdehyde; PC—control group receiving a mineral and vitamin premix containing (per kg of feed): 20 mg CuSO_4_, 100 mg ZnO and 100 mg MnO; IR—group receiving a mineral and vitamin premix containing (per kg of feed): 2 mg CuSO_4_, 10 mg ZnO and 10 mg MnO; NR—group receiving a mineral and vitamin premix containing (per kg of feed): 2 mg NP-Cu, 10 mg NP-ZnO and 10 mg NP-Mn_2_O_3_; NC—group receiving a mineral and vitamin premix without Cu, Zn, and Mn.

**Table 10:** Indicators of redox status in the bursa of Fabricius in turkeys.

Item	PC	IR	NR	NC	SEM	*p*-Value
SOD U/g protein	14.82 ^a^	10.19 ^b^	6.74 ^c^	6.12 ^c^	0.812	<0.001
CAT U/g protein	38.29	39.84	38.00	37.67	0.383	0.193
GSH+GSSG µmol/kg	0.746	0.704	0.683	0.678	0.014	0.300
VIT C µmol/kg	202.34	231.37	226.93	236.14	5.096	0.080
LOOH µmol/ kg	6.99	6.96	7.06	7.04	0.021	0.317
MDA µmol/kg	5.52	6.48	6.97	6.13	0.256	0.243

a, b, c—means within the same row differ significantly (*p* ≤ 0.05); SOD—superoxide dismutase; CAT—catalase; GSH+GSSG—total glutathione; VIT C—vitamin C; LOOH—lipid peroxides; MDA—malondialdehyde; PC—control group receiving a mineral and vitamin premix containing (per kg of feed): 20 mg CuSO_4_, 100 mg ZnO and 100 mg MnO; IR—group receiving a mineral and vitamin premix containing (per kg of feed): 2 mg CuSO_4_, 10 mg ZnO and 10 mg MnO; NR—group receiving a mineral and vitamin premix containing (per kg of feed): 2 mg NP-Cu, 10 mg NP-ZnO and 10 mg NP-Mn_2_O_3_; NC—group receiving a mineral and vitamin premix without Cu, Zn, and Mn.

## References

[1] Richards J.D., Zhao J., Harrell R.J., Atwell C.A., Dibner J.J. (2010). Trace mineral nutrition in poultry and swine. Asian-Aust. J. Anim. Sci..

[2] Olgun O. (2017). Manganese in poultry nutrition and its effect on performance and eggshell quality. Worlds Poult. Sci. J..

[3] Kozłowski K., Jankowski J., Otowski K., Zduńczyk Z., Ognik K. (2018). Metabolic parameters in young turkeys fed diets with different inclusion levels of copper nanoparticles. Pol. J. Vet. Sci..

[4] Ognik K., Sembratowicz I., Cholewińska E., Jankowski J., Kozłowski K., Juśkiewicz J., Zduńczyk Z. (2018). The effect of administration of copper nanoparticles to chickens in their drinking water on the immune and antioxidant status of the blood. Anim. Sci. J..

[5] Ognik K., Kozłowski K., Stępniowska A., Szlązak R., Tutaj K., Zduńczyk Z., Jankowski J. (2019). The effect of manganese nanoparticles on performance, redox reactions and epigenetic changes in turkey tissues. Animal.

[6] Jankowski J., Ognik K., Stępniowska A., Zduńczyk Z., Kozłowski K. (2018). The effect of manganese nanoparticles on apoptosis and on redox and immune status in the tissues of young turkeys. PLoS ONE.

[7] Jankowski J., Ognik K., Stępniowska A., Zduńczyk Z., Kozłowski K. (2019). The effect of the source and dose of manganese on the performance, digestibility and distribution of selected minerals, redox, and immune status of turkeys. Poult. Sci..

[8] Bao Y.M., Choct M. (2009). Trace mineral nutrition for broiler chickens and prospects of application of organically complexed trace minerals: A review. Anim. Prod. Sci..

[9] Świątkiewicz S., Arczewska-Włosek A., Józefiak D. (2014). The efficacy of organic minerals in poultry nutrition: Review and implications of recent studies. World Poult. Sci. J..

[10] Scott A., Vadalasetty K.P., Chwalibog A., Sawosz E. (2018). Copper nanoparticles as an alternative feed additive in poultry diet: A review. Nanotechnol. Rev..

[11] Saripinar Aksu D., Aksu T., Ozsoy B., Baytok E. (2010). The effects of replacing inorganic with a lower level of organically complexed minerals (Cu, Zn, and Mn) in broiler diets on lipid peroxidation and antioxidant defense systems. Asian-Aust. J. Anim. Sci..

[12] Oliveira T.F.B., Bertechini A.G., Bricka R.M., Kim E.J., Gerard P.D., Peebles E.D. (2015). Effects of in ovo injection of organic zinc, manganese, and copper on the hatchability and bone parameters of broiler hatchlings. Poult. Sci..

[13] National Research Council (NRC) (1994). Nutrient Requirements of Poultry.

[14] British United Turkeys, Aviagen Turkeys (2013). Management Guidelines for Raising Commercial Turkeys. https://www.aviagenturkeys.com/media/183481/aviagencommercialguide.pdf.

[15] Hybrid Commercial Nutrient Guidelines. https://www.hybridturkeys.com/en/resources/commercial-management.

[16] European Commission (2003). Opinion of the Scientific Committee for Animal Nutrition on the Use of Copper in Feeding Stuffs. https://echa.europa.eu/documents/10162/13630/vrar_appendix_e_en.pdf/5ff87011-605c-434e-a661-7a687da97bf5.

[17] (2018). Commission Implementing Regulation (EU) 2018/1039. concerning the authorisation of Copper(II) diacetate monohydrate, Copper(II) carbonate dihydroxy monohydrate, Copper(II) chloride dihydrate, Copper(II) oxide, Copper(II) sulphate pentahydrate, Copper(II) chelate of amino acids hydrate, Copper(II) chelate of protein hydrolysates, Copper(II) chelate of glycine hydrate (solid) and Copper(II) chelate of glycine hydrate (liquid) as feed additives for all animal species and amending Regulations (EC) No 1334/2003, (EC) No 479/2006 and (EU) No 349/2010 and Implementing Regulations (EU) No 269/2012, (EU) No 1230/2014 and (EU) 2016/2261.

[18] Collins N.E., Moran E.T. (1999). Influence of supplemental manganese and zinc on live performance and carcass quality of broilers. J. Appl. Poult. Res..

[19] Suttle N.F. (2010). The Mineral Nutrition of Livestock.

[20] Ognik K., Stępniowska A., Cholewińska E., Kozłowski K. (2016). The effect of administration of copper nanoparticles to chickens in drinking water on estimated intestinal absorption of iron, zinc, and calcium. Poult. Sci..

[21] Dusek P., Jankovic J., Le W. (2012). Iron dysregulation in movement disorders. Neurobiol. Dis..

[22] Du Y., Zhu Y.H., Teng X.J., Zhang K., Teng X.H., Li S. (2015). Toxicological effect of manganese on NF-κB/iNOS-COX-2 signaling pathway in chicken testes. Biol. Trace Elem. Res..

[23] Aksu T., Aksu M.I., Yoruk M.A., Karaoglu M. (2011). Effects of organically complexed minerals on meat quality in chickens. Br. Poult. Sci..

[24] EFSA Panel on Additives and Products or Substances used in Animal Feed (FEEDAP) (2016). Revision of the currently authorised maximum copper content in complete feed. EFSA J..

[25] Sri Sindhura K., Prasad T.N.V.K.V., Panner Selvam P., Hussain O.M. (2014). Synthesis, characterization and evaluation of effect of phytogenic zinc nanoparticles on soil exo-enzymes. Appl. Nanosci..

[26] Ognik K., Wertelecki T. (2012). Effect of different vitamin E sources and levels on selected oxidative status indices in blood and tissues as well as on rearing performance of slaughter turkey hens. J. Appl. Poult. Res..

[27] Yang X.J., Sun X.X., Li C.Y., Wu X.H., Yao J.H. (2011). Effects of copper, iron, zinc, and manganese supplementation in a corn and soybean meal diet on the growth performance, meat quality, and immune responses of broiler chickens. J. Appl. Poult. Res..

[28] El-Husseiny O.M., Hashish S.M., Ali R.A., Arafa S.A., Abd El- Samee L.D., Olemy A.A. (2012). Effects of Feeding Organic Zinc, Manganese and Copper on Broiler Growth, Carcass Characteristics, Bone Quality and Mineral Content in Bone, Liver and Excreta. Int. J. Poult. Sci..

[29] Gheisari A.A., Rahimi-Fathkoohi A., Toghyani M., Gheisari M.M. (2010). Effects of organic chelates of zinc, manganese and copper in comparison to their inorganic sources on performance of broiler chickens. J. Anim. Plant Sci..

[30] Fathi M. (2016). Effects of Zinc Oxide Nanoparticles Supplementation on Mortality due to Ascites and Performance Growth in Broiler Chickens. Iran. J. Appl. Anim. Sci..

[31] Jankowski J., Kozłowski K., Ognik K., Otowski K., Juśkiewicz J., Zduńczyk Z. (2019). The effect of the dietary inclusion levels and sources of zinc on the performance, metabolism, redox and immune status of turkeys. Anim. Feed Sci. Technol..

[32] Wang X., Fosmire G.J., Gay C.V., Leach R.M. (2002). Short term zinc deficiency inhibits chondrocyte proliferation and induced cell apoptosis in the epiphyseal growth plate of young chickens. J. Nutr..

[33] Azad S.K., Shariatmadari F., Torshizi M.A.K., Ahmadi H. (2017). Effect of zinc concentration and source on performance, tissue mineral status, activity of superoxide dismutase enzyme and lipid peroxidation of meat in broiler chickens. Anim. Prod. Sci..

[34] Bao M.Y., Choct M., Iji P.A., Bruerton K. (2007). Effect of organically complexed copper, iron, manganese, and zinc on broiler performance, mineral excretion and accumulation in tissues. J. Appl. Poult. Res..

[35] Berta E., Andrásofszky E., Bersényi A., Glávits R., Gáspárdy A., Fekete S.G. (2004). Effect of inorganic and organic manganese supplementation on the performance and tissue manganese content of broiler chicks. Acta Vet. Hung..

[36] Cholewińska E., Ognik K., Fotschki B., Zduńczyk Z., Juśkiewicz J. (2018). Comparison of the effect of dietary copper nanoparticles and one copper (II) salt on the copper biodistribution and gastrointestinal and hepatic morphology and function in a rat model. PLoS ONE.

[37] Cholewińska E., Fotschki B., Juśkiewicz J., Rusinek-Prystupa E., Ognik K. (2018). The effect of copper level in the diet on the distribution, and biological and immunological responses in a rat model. J. Anim. Feed Sci..

[38] Cholewińska E., Juśkiewicz J., Ognik K. (2018). Comparison of the effect of dietary copper nanoparticles and one copper (II) salt on the metabolic and immune status in a rat model. J. Trace Elem. Med. Biol..

[39] Gajula S.S., Chelasani V.K., Panda A.K., Mantena V.L., Savaram R.R. (2011). Effect of Supplemental Inorganic Zn and Mn and their Interactions on the Performance of Broiler Chicken, Mineral Bioavailability, and Immune Response. Biol. Trace. Elem. Res..

[40] Sunder G.S., Panda A.K., Gopinath N.C.S., Rama Rao S.V., Raju M.V.L.N., Reddy M.R., Vijaya Kumar C. (2008). Effects of higher levels of zinc supplementation on performance, mineral availability, and immune competence in broiler chickens. J. Appl. Poult. Res..

[41] Cao H., Su R., Hu G., Li C., Guo J., Pan J., Tang Z. (2016). In vivo effects of high dietary copper levels on hepatocellular mitochondrial respiration and electron transport chain enzymes in broilers. Br. Poult. Sci..

[42] Min L., Wei C., Xi P., Cai Min B., Heng Min C. (2009). Effect of dietary high copper on the oxidization in brain tissue of chickens. Chin. J. Vet. Sci..

[43] Oguz E.O., Enli Y., Tufan A.C., Turgut G. (2014). Toxic effects of copper sulfate on the brains of term Hubbard broiler chicks: A stereological and biochemical study. Biotech. Histochem..

